# Case Report: *CD40LG* Arg203Ile variant underlies atypical phenotype of X-linked hyper IgM syndrome

**DOI:** 10.3389/fimmu.2025.1572791

**Published:** 2025-05-05

**Authors:** Takuro Nishikawa, Dan Tomomasa, Atsushi Hijikata, Hiroshi Kasabata, Yasuhiro Okamoto, Hans D. Ochs, Hirokazu Kanegane

**Affiliations:** ^1^ Department of Pediatrics, Graduate School of Medical and Dental Sciences, Kagoshima University, Kagoshima, Japan; ^2^ Department of Pediatrics and Developmental Biology, Graduate School of Medical and Dental Sciences, Institute of Science Tokyo, Tokyo, Japan; ^3^ School of Life Sciences, Tokyo University of Pharmacy and Life Sciences, Tokyo, Japan; ^4^ Department of Clinical Laboratory Medicine, Kagoshima University Hospital, Kagoshima, Japan; ^5^ Department of Pediatrics, University of Washington, and Seattle Children’s Research Institute, Seattle, WA, United States; ^6^ Department of Child Health and Development, Graduate School of Medical and Dental Sciences, Institute of Science Tokyo, Tokyo, Japan

**Keywords:** atypical phenotype, CD40 ligand, immunoglobulin replacement therapy, structural stability assessment, x-linked hyper IgM syndrome

## Abstract

Hyper IgM syndrome (HIGM) is a rare immunodeficiency caused by impaired immunoglobulin class switching, leading to recurrent infections. The present report describes the case of an 18-year-old man initially diagnosed with common variable immunodeficiency at 3 years of age. Genetic analysis revealed a hemizygous *CD40LG* missense variant (p.Arg203Ile) associated with X-linked HIGM (XHIGM). Structural and flow cytometric analyses indicated normal CD40 ligand (CD40L) expression on activated CD4^+^ T-cells but impaired CD40 binding, indicating disrupted immune signaling. Notably, the patient experienced neither bacterial infections requiring hospitalization nor opportunistic infections during 15 years of immunoglobulin replacement therapy. These findings indicate that the p.Arg203Ile variant destabilizes CD40L–CD40 interactions without affecting CD40L expression, suggesting a hypomorphic phenotype. This report highlights the importance of combining genetic testing with functional analysis when evaluating atypical XHIGM presentations to predict clinical severity and provide a scientific basis for personalized treatment strategies. Additional studies are required to assess the long-term outcomes and potential curative therapies for similar cases.

## 1 Introduction

Hyper IgM syndromes (HIGM), of which eight molecularly defined forms are known, are inborn errors of immunity characterized by increased susceptibility to infection due to impaired immunoglobulin class switching (1, 2). The most frequent HIGM category, X-linked HIGM (XHIGM) or CD40 ligand (CD40L) deficiency, arises from a defect in CD40L caused by *CD40LG* variants. XHIGM is a complex immunodeficiency, often associated with opportunistic infections, such as *Pneumocystis jirovecii* pneumonia and *Cryptosporidium* enteritis, and bacterial infections, resulting from T-cell dysfunction and impaired antibody production. In addition to infection prevention and management, immune reconstitution through allogeneic hematopoietic stem cell transplantation (HSCT) offers a curative option (1, 2).

CD40L, a member of the tumor necrosis factor superfamily, interacts with its receptor CD40, which is constitutively expressed in B-cells, resulting in B-cell maturation and immunoglobulin class switching. Pathogenic *CD40LG* variants result in absent or nonfunctional CD40L expression on activated CD4^+^ T-cells. This disrupts its interaction with CD40 expressed by macrophages and dendritic cells. This disruption prevents the generation of costimulatory immune responses essential for T-cell differentiation. Consequently, patients with XHIGM exhibit impaired humoral and cellular immunity and are vulnerable to opportunistic and recurrent bacterial infections (1, 2).

In the present report, we describe a case of delayed diagnosis of XHIGM in an 18-year-old man who received a diagnosis of common variable immunodeficiency (CVID) at 3 years of age. Using target gene panel sequencing, we identified a missense variant (p.Arg203Ile) in *CD40LG*, which resulted in an atypical XHIGM phenotype.

## 2 Methods

### 2.1 Ethics statement

This study was conducted in accordance with the principles of the Declaration of Helsinki and was approved by the Ethics Committee on Clinical Research of the Sakuragaoka Campus, Kagoshima University (No. 240122). Informed consent was obtained from the patient and his parents for participation in this study and the publication of the data and images.

### 2.2 Genetic analysis

Target gene panel sequencing of relevant HIGM genes, namely *CD40LG*, *AICDA*, *CD40*, *UNG*, *INO80*, *PIK3CD*, *PIK3R1*, *PTEN*, and *IKBKG*, was performed using genomic DNA isolated from the whole blood of both the patient and his mother. The variant was validated using Sanger sequencing.

### 2.3 T-cell activation, detection of CD40LG expression, and CD40-Ig binding assay using flow cytometry

Peripheral blood mononuclear cells were stimulated for 4 h at 37°C in the presence of 10 ng/mL phorbol 12-myristate 13-acetate and 1 µg/mL ionomycin before staining with the following antibodies: FITC-conjugated anti-CD3 (BD Biosciences, Franklin Lakes, NJ, USA), PerCP-conjugated anti-CD69 (BD Biosciences), APC-conjugated anti-CD4 (Beckman Coulter, Brea, CA, USA), PacificBlue-conjugated anti-CD8 (Beckman Coulter), and anti-CD40L/CD154 (**Supplementary Table 1**). After staining, CD3^+^, CD4^+^, CD8-, and CD69^+^ cells were gated and analyzed. For the CD40-Ig binding assay, either clone 89-76 anti-CD40L/CD154 antibody or CD40-muIg/R-PE (Ancell Corporation, Minneapolis, MN, USA) was used. Subsequently, the ratio of cells expressing CD154 to those expressing CD40-Ig was determined to estimate the binding capacity of CD40 (3). Flow cytometry was performed using a BD LSR Fortessa™ X-20 (BD Biosciences), and data were analyzed using the FlowJo software (BD Biosciences).

## 3 Case description

The patient was hospitalized four times for acute bronchitis at the age of 2 and acute pneumonia at the ages of 2 years and 6 months, 3 years and 7 months, and 3 years and 8 months, during which he received antimicrobial treatment. Therefore, the patient was referred to our hospital. He exhibited normal development and growth, and his family medical history was otherwise unremarkable. His white blood cell count was 10190/μL, with 34% granulocytes and 56.5% lymphocytes (5757/μL). His serum IgG level was low, whereas his IgA and IgM levels were elevated (306, 534, and 245 mg/dL, respectively; normal age-based ranges: 638–1536, 50–254, and 22–216 mg/dL). His IgE level was 149 IU/mL (normal value: ≤ 20 IU/mL). He had received the measles vaccine; however, the measles antibody test was negative. Lymphocyte subpopulation analysis revealed the following cell counts: CD3^+^ T-cells, 4087/μL (normal age-based range: 1794–4247/μL); NK cells, 288/μL (270–1053/μL); and CD19^+^ B-cells, 1267/μL (461–1456/μL). Although CD19^+^ B-cell numbers were within the normal range, those of CD27^+^ memory CD19^+^ B-cells were reduced, constituting 6.7% of the CD19^+^ cell population. Naïve CD8^+^ and CD4^+^ T-cells were within the normal range, accounting for 67% of the CD3^+^CD4^+^ cell population and 89.1% of the CD3^+^CD8^+^ cell population, respectively. *In vitro* lymphocyte stimulation with phytohemagglutinin and concanavalin A were unremarkable (58316 cpm, normal range: 41000–79900 cpm; and 30043 cpm, normal range: 20300–65700 cpm). Based on these findings, CVID was diagnosed, and intravenous immunoglobulin replacement (400 mg/kg every 4 weeks) was initiated to prevent infections. At 8 years of age, the patient was switched to weekly subcutaneous immunoglobulin injections. After initiating immunoglobulin replacement therapy, the patient experienced no bacterial infections requiring hospitalization. To date, the patient has had no episodes, laboratory data, or chest X-ray findings suggestive of lung or liver disease.

**Figure 1:**
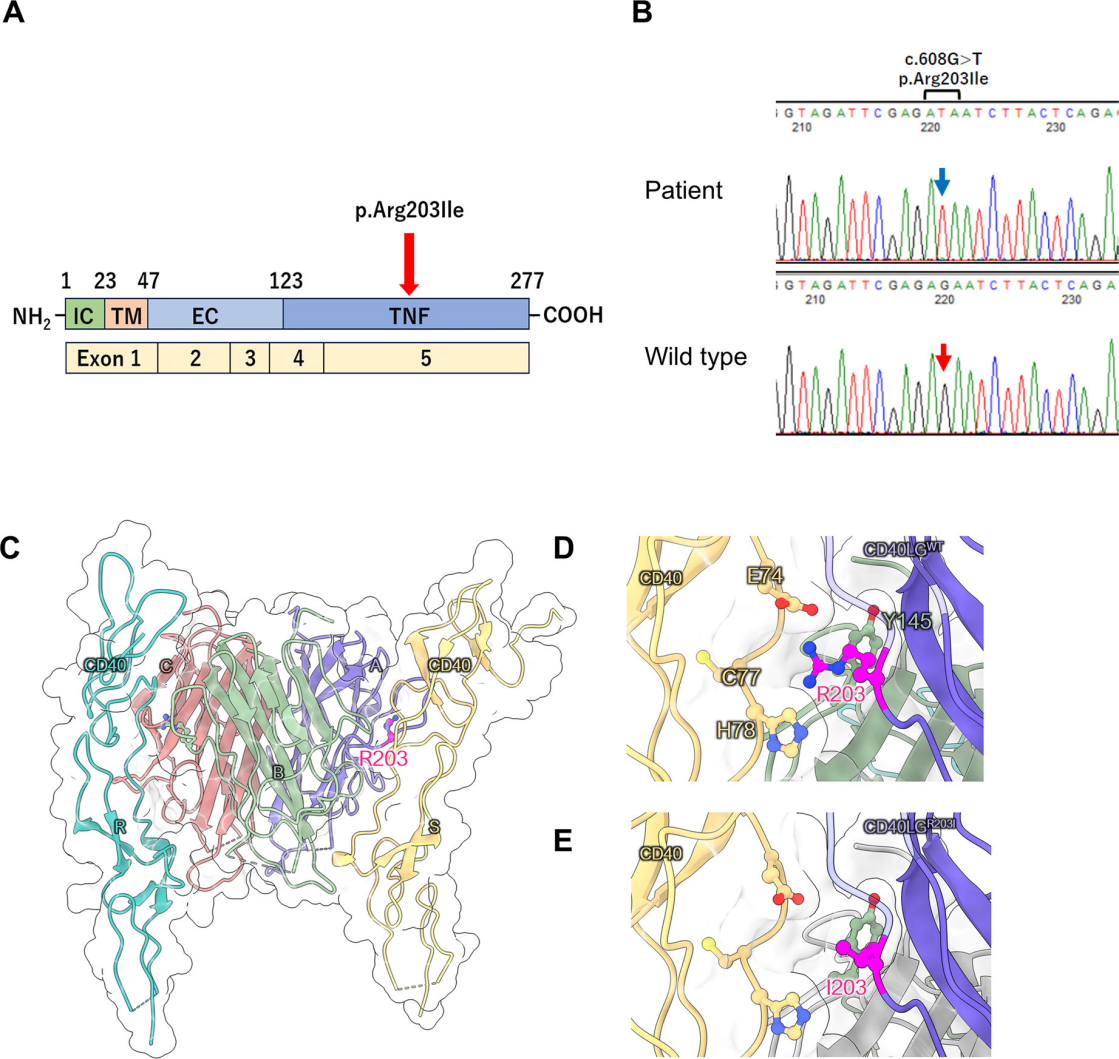
**(A)** Location of the *CD40LG* variant in the patient with X-linked hyper IgM syndrome. Schematic representation of the four domains of the CD40 ligand protein and five exons of *CD40LG*. IC, intracellular tail; TM, transmembrane domain; ECU, extracellular unique region; TNF, tumor necrosis factor homology domain. **(B)** Sanger sequencing of *CD40LG*. A hemizygous variant (c.608G>T) was identified in the genomic DNA isolated from peripheral blood mononuclear cells of the patient. The blue arrow indicates the position of c.608T in the *CD40LG* variant, while the red arrow indicates the c.608G position in wild-type *CD40LG*. **(C–E)** Structural analysis of CD40 ligand protein. **(C)** Structure of the CD40 ligand trimer and CD40 dimer complex (PDB code: 3QD6). R203 in CD40 ligand (chains A and B) localizes to the CD40 interface (chains S and R, respectively). **(D, E)** Enlarged views of the local structure of R203 and its substitution with I203.

At 18 years of age, the patient underwent targeted panel sequencing of HIGM-related genes— (*CD40LG*, *AICDA*, *CD40*, *UNG*, *INO80*, *PIK3CD*, *PTEN*, and *IKBKG—* at the Kazusa DNA Research Institute using genomic DNA from peripheral blood mononuclear cells. A hemizygous missense variant (c.608G>T, p.Arg203Ile) was identified in *CD40LG*. **Figure 1A** illustrates the location of the *CD40LG* variant in this patient. **Figure 1B** depicts the results of Sanger sequencing. *In silico* analysis of the c.608G>T *CD40LG* variant revealed a minor allele frequency of –6 and a Combined Annotation-Dependent Depletion score of 24.9 (**Supplementary Figure 1**). Amino acid sequence alignment of CD40L revealed that the position of this missense variant (p.Arg203Ile) is conserved across all species, highlighting its functional importance (**Supplementary Table 2**). Moreover, the atomic coordinates of the crystal structure of the CD40L–CD40 complex were obtained from the Protein Data Bank (PDB code: 3QD6). In this structure, CD40L formed a homotrimer that bound two CD40 molecules, with the Arg203 residues being located on the CD40 interface and forming electrostatic interactions with Glu74 of CD40 (**Figures 1C–E**). The replacement of Arg with Ile was predicted to disrupt the CD40L–CD40 interaction. Thus, we evaluated the effect of the p.Arg203Ile variant on the thermal stability of the CD40L–CD40 complex structure using the FoldX software version 4 (4). As expected, the computed folding ΔΔG values for each *CD40L* protomer were small. However, the binding ΔΔG values of the CD40L homotrimer to each CD40 were +1.90 and +2.19 kcal/mol, respectively. This finding suggests that p.Arg203Ile destabilizes the interaction with CD40 rather than the overall structural stability of CD40L (**Supplementary Tables 3**, **4**). Consistent with the finding, we obtained a similar result using the mCSM web-based structural bioinformatics tool (5), which predicts changes in binding affinity induced by a given variant. Using this approach, we confirmed that p.Arg203Ile destabilizes the CD40L–CD40 interaction (**Supplementary Figure 2**).

**Figure 2:**
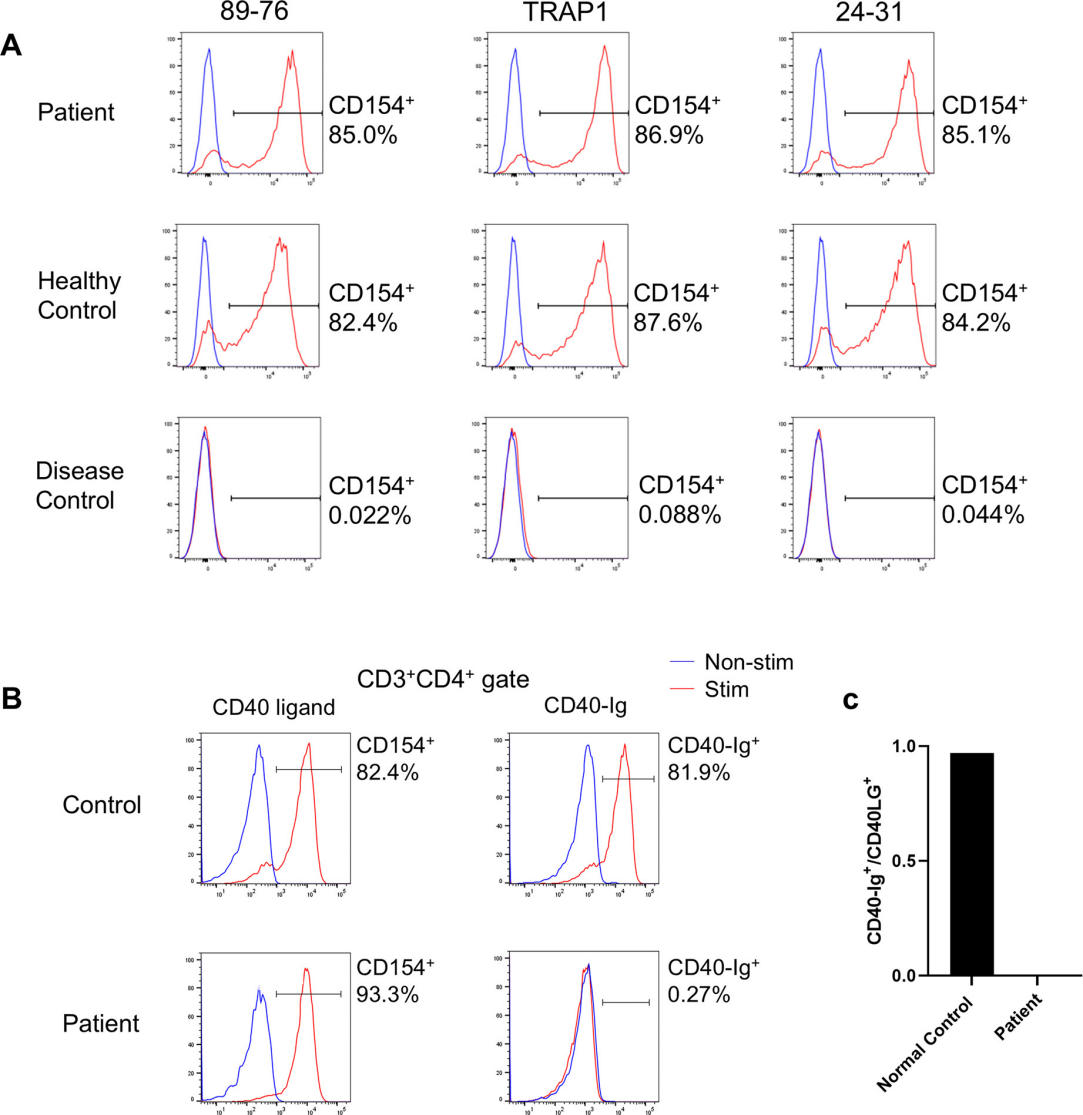
**(A)** CD40 ligand expression on activated CD4^+^ T-cells. Flow cytometry analysis using three different anti-CD40 ligand/CD154 antibodies (89-76, TRAP1, and 24-31) revealed no decrease in binding by the activated T-cells (CD4^+^CD154^+^) of the patient compared to those of a healthy control. In contrast, activated CD4^+^ T-cells from a patient with X-linked hyper IgM syndrome with typical phenotype (disease control) did not bind to any anti-CD40L/CD154 antibodies. CD40L expression by CD4^+^ T cells after phorbol 12-myristate 13-acetate (PMA) and ionomycin stimulation (red), unstimulated cells (blue). **(B)** CD40 ligand binding to CD40-Ig on activated CD4^+^ T-cells. Flow cytometry comparing anti-CD40L/CD154 antibody 89-76 binding (left panel) with CD40-Ig (CD40-muIg/R-PE) binding (right panel). Functional CD40L expressed by CD4^+^ T-cells following PMA and ionomycin stimulation (red) and by unstimulated cells (blue). While patient cells exhibited comparable anti-CD40L/CD154 antibody binding to control cells, they demonstrated no CD40-Ig construct binding. **(C)** Percentage of CD3^+^CD154^+^ cells that bind CD40-Ig. The ratio of CD3^+^CD40-Ig^+^:CD3^+^CD154^+^ cells (×100) post-PMA and ionomycin stimulation determines the percentage of CD3^+^CD154^+^ cells binding to CD40-Ig. While control cells exhibited over 90% binding, patient cells exhibited nearly zero CD40-Ig binding.

Flow cytometry using three different anti-CD40L/CD154 antibodies (89-76, TRAP1, 24-31; **Supplementary Table 1**) revealed no reduction in CD40L^+^ activated T-cell (CD154^+^) levels compared to those of the controls (**Figure 2A**). However, we found that this variant interferes with the ability of the CD40L variant to bind to CD40. Specifically, in patient cells, only 0.27% of CD3+CD4+ cells were able to bind soluble CD40-Ig, whereas a significant proportion (81.9%) of control CD3^+^CD4^+^ cells were able to bind it (**Figures 2B, C**). Based on these results, the diagnosis was revised to XHIGM with an atypical phenotype. The father and mother of the patient were healthy and had no significant medical history, necessitating no immunological tests. The healthy mother of the patient was found to be a carrier of this heterozygous missense variant.

## 4 Discussion

Mild phenotypes have been reported in 6 of 77 (7.8%) unrelated patients with XHIGM (6). One patient harbored a nonsense variant (c.31C>T, p.Arg11stop), which enables transcription initiation at the next methionine site at position 21, resulting in a protein lacking the cytoplasmic domain. This explains the binding of both anti-CD40L/CD154 antibody and CD40Ig. Another patient exhibited an in-frame deletion in exon 2 caused by a splice-site variant (IVS2 + 2t>a), allowing small amounts of wild-type mRNA to be generated. A third patient carried a “silent” point variant (367G>A) affecting the last nucleotide of exon 3, leading to a splicing defect resulting in exon 3 skipping. However, small amounts of normally spliced wild-type mRNA were also generated. Moreover, three mild cases from unrelated families demonstrated the same amino acid substitution (p.Thr254Met) affecting the carboxyl-terminal of exon 5, located six amino acids upstream from the termination codon (6). The same missense variant was identified in three brothers from Argentina who exhibited clinical and immunological features indistinguishable from the rest of the cohort (n = 11) (7). Overall, hypomorphic variants that permit the CD40L– CD40 interaction are frequently associated with an atypical and milder clinical course (6, 8, 9). Notably, hypomorphic variants in the *CD40L* transmembrane domain may result in reduced but functional CD40L expression, with affected patients often exhibiting relatively mild clinical features (8, 9). Although another patient with XHIGM with the p.Arg203Ile variant has been previously reported, neither the clinical course nor treatment outcome was described (6). However, the findings of this previous report aligned with the present report, indicating normal CD40L expression but absent CD40-Ig binding. Furthermore, our structural analysis and CD40-Ig binding assay indicated that p.Arg203Ile does not affect CD40L structure stability but significantly impairs its CD40 interaction. This finding is consistent with a recent study demonstrating that XHIGM-related variants at the interface, such as p.Arg207Ala and p.Lys143Ala, significantly suppress the CD40–CD40L interaction (10).

As HSCT remains the only curative treatment for XHIGM, it is recommended to perform HSCT before the age of 10 years and before any organ damage (11, 12). However, infection control was maintained in our patient through consistent immunoglobulin replacement therapy alone. Our patient never developed opportunistic infections, such as *Pneumocystis jiroveci*, fungal, or *Cryptosporidium* infections. Therefore, further studies are needed to assess long-term outcomes and potential curative therapies for similar cases. As our patient experienced only mild infections after immunoglobulin replacement was initiated at age three years, a comprehensive genetic evaluation at that time was not performed. Despite low IgG and high IgM/IgA levels, along with the male sex—which should have suggested XHIGM—a presumptive CVID diagnosis was made. The mild clinical phenotype of our patient, caused by the p.Arg203Ile variant but resembling CVID, suggested a predominant antibody deficiency without apparent T-cell dysfunction.

One limitation of this study was the use of target gene panel sequencing for HIGM-related genes rather than whole-exome sequencing, potentially overlooking the possible contributions of other genetic variants to the atypical phenotype. However, we believe that the *CD40LG* Arg203Ile variant explains the current phenotype; therefore, it is unlikely that additional variants contributing to this phenotype could have been identified through exome sequencing. Our thermal stability studies using structural bioinformatics software, along with the previous study on a patient with XHIGM carrying the same missense variant (6), provide valuable insights into the destabilizing effect of this variant on the CD40L–CD40 interaction.

In conclusion, this report demonstrates that assessing *CD40LG* genetic variants and their effects on CD40L expression and function may provide useful information for guiding prognostic and therapeutic decisions.

## Data Availability

The datasets for this article are not publicly available due to concerns regarding participant/patient anonymity. Requests to access the datasets should be directed to the corresponding author.
