# Supplementary material for: Case Report: CD40LG Arg203Ile variant underlies atypical phenotype of X-linked hyper IgM syndrome
Source: Front Immunol. 2025 May 5;16:1572791. doi: 10.3389/fimmu.2025.1572791 (PMC12086146; doi:10.3389/fimmu.2025.1572791)
Supplement: Supplementary file 1 [file Table1.docx]

Supplementary Material

# Supplementary Figures and Tables

## Supplementary Figures


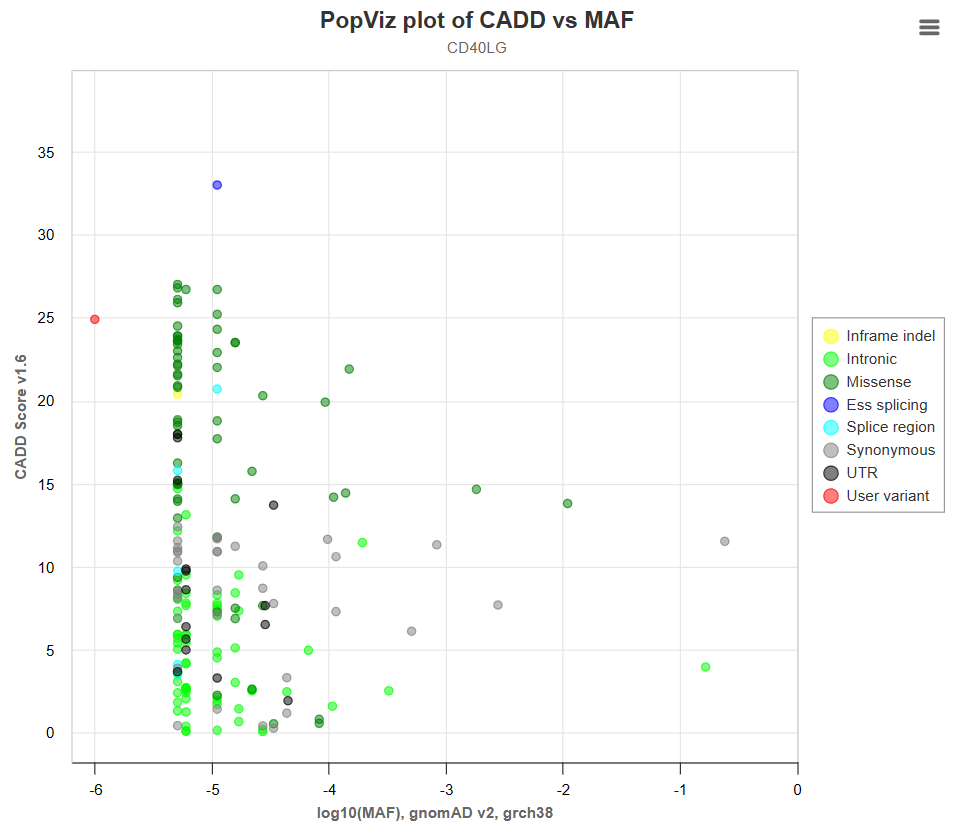


**Supplementary Figure 1. *In silico* analysis of the *CD40LG* (c.608G>T) variant.** MAF and CADD scores of *CD40L* using PopViz. The scores were -6 and 24.9 for the c.608G>T variant, respectively.

## Supplementary Tables

**Supplementary Table 1. CD40L/CD154 antibodies used for flow cytometry.**

| **Clone** | **Product name** | **Isotype** | **Company** |
| --- | --- | --- | --- |
| 89-76 | BD™  PE mouse anti-human CD154 | Mouse (BALB/c) IgG1, κ | BD Biosciences |
| TRAP1 | CD154(CD40L)-PE | IgG1 mouse | Beckman Coulter |
| 24-31 | BD Pharmingen™  PE mouse anti-human CD40L (CD154) | Mouse (BALB/c) BALB/c IgG1, κ | BD Pharmingen |

**Supplementary Table 2. Amino acid sequence alignment of CD40L among various species**.

| Species | 201 | 202 | 203 | 204 | 205 |
| --- | --- | --- | --- | --- | --- |
| *Homo sapiens* | F | E | R | I | L |
| *Pan troglodytes* | F | E | R | I | L |
| *Macaca mulatta* | F | E | R | I | L |
| *Canis lupus* | T | E | R | V | L |
| *Bos taurus* | S | E | R | I | L |
| *Mus musculus* | S | E | R | I | L |
| *Rattus norvegicus* | S | E | R | I | L |
| *Gallus gallus* | E | D | R | L | L |
| *Xenopus tropicalis* | T | D | K | L | L |

The red color indicates the position of the missense variant (p.Arg203Ile).

**Supplementary Table 3. Changes in the thermal stability of the CD40L monomer**

| Protein | ΔΔG (kcal/mol) |
| --- | --- |
| CD40L^WT^ vs. CD40L^R203I^ (chain A^*^) | -0.97 (± 0.04) |
| CD40L^WT^ vs. CD40L^R203I^ (chain B^*^) | -0.20 (± 0.08) |
| CD40L^WT^ vs. CD40L^R203I^ (chain C^*^) | -1.05 (± 0.15) |

The predicted folding ΔΔG of the CD40L p.Arg203Ile variant for each protomer was -0.97, -0.20, and -1.05 kcal/mol, respectively. ^*^The chain IDs in parentheses indicate designated identifiers for each polypeptide chain in the PDB entry (PDB code: 3qd6).

**Supplementary Table 4. CD40L vs CD40 complex interaction in response to the missense variant (p.Arg203Ile).**

| Interaction pair | ΔΔG_bind_ (kcal/mol) |
| --- | --- |
| CD40L (chain A^*^) vs. CD40 (chain S^*^) | 1.90 (± 0.49) |
| CD40L (chain C^*^) vs. CD40 (chain R^*^) | 2.19 (± 0.44) |

The computed binding ΔΔG of the CD40L (p.Arg203Ile) variant to each CD40 was +1.90 and +2.19 kcal/mol, respectively. ^*^The chain IDs in parentheses indicate designated identifiers for each polypeptide chain in the PDB entry (PDB code: 3qd6).
